# Antifungal Activity and Transcriptomic Profiling of Equisetin from Endophytic *Fusarium incarnatum* Y2 Against Major Wheat Root and Crown Rot Pathogens

**DOI:** 10.3390/genes17080869

**Published:** 2026-07-25

**Authors:** Miao Liu, Feifan Wang, Luying Han, Feiyu Yan, Yinshan Huang, Yuehua Geng, Chunnan Wen, Luyang Song, Meng Zhang, Fang Liu, Qingzhou Ma

**Affiliations:** 1 College of Agronomy, Henan Agricultural University, Zhengzhou 450046, China; liumiao@henau.edu.cn (M.L.); w13949147723@163.com (F.W.); 18838280398@163.com (L.H.); xhh1479@163.com (Y.H.); cnwen@henau.edu.cn (C.W.); 2 Zhengzhou Key Laboratory of Germplasm Resources Innovation and Metabolic Engineering of Traditional Chinese Medicine, Henan Agricultural University, Zhengzhou 450046, China; 3 College of Plant Protection, Henan Agricultural University, Zhengzhou 450046, China; 17630711375@163.com (F.Y.); gyh@henau.edu.cn (Y.G.); lysong@henau.edu.cn (L.S.); zm2006@126.com (M.Z.); 4 State Key Laboratory of High-Efficiency Production of Wheat-Maize Double Cropping, Henan Agricultural University, Zhengzhou 450002, China; 5 College of Resources and Environment, Henan Agricultural University, Zhengzhou 450046, China

**Keywords:** *Fusarium incarnatum*, endophytic fungi, equisetin, biocontrol, wheat pathogens, transcriptomics

## Abstract

**Background/Objectives**: Wheat root and crown rot, caused by *Fusarium pseudograminearum*, *Fusarium graminearum*, and *Bipolaris sorokiniana*, are devastating soil-borne diseases that cause substantial yield losses worldwide. Endophytic fungi are promising sources of bioactive metabolites for agricultural applications. This study aimed to isolate and characterize an endophytic fungus with antifungal activity against major wheat pathogens, identify its active compound, and investigate the underlying transcriptional response. **Methods**: An endophytic strain Y2 was isolated from *Hedyotis diffusa* leaves and identified through morphological and phylogenetic analysis based on *TEF-1α* and *RPB2* sequences. Pathogenicity of strain Y2 was evaluated on wheat stem bases. The bioactive compound was purified by HPLC and identified by HR-ESI-MS and NMR. Antifungal activity was assessed using dual-culture and microbroth dilution assays. Transcriptomic analysis (RNA-seq) was performed on *F. pseudograminearum* treated with equisetin, with qRT-PCR validation of seven representative differentially expressed genes. **Results**: Strain Y2 was identified as *Fusarium incarnatum* or a closely related member of the *F. incarnatum–equiseti* species complex (FIESC) and confirmed to be non-pathogenic to wheat. The purified bioactive compound was characterized as equisetin, which exhibited significant antifungal activity with MIC values of 16, 32, and 64 μg/mL against *F. pseudograminearum*, *B. sorokiniana*, and *F. graminearum*, respectively. Transcriptomic analysis revealed that equisetin treatment induced a polarized transcriptional response in *F. pseudograminearum*, characterized by strong upregulation of ribosome and translation-related genes and widespread downregulation of other metabolic pathways, particularly nitrogen metabolism. qRT-PCR validation of seven representative genes confirmed the reliability of the RNA-seq data. **Conclusions**: Our findings demonstrate that equisetin is the active antifungal metabolite produced by *F. incarnatum* Y2, with potent in vitro activity against major wheat root and crown rot pathogens. The transcriptomic data provide insights into the potential mechanism of action, while the non-pathogenic nature of strain Y2 supports its biosafety. Although these results highlight equisetin as a promising lead compound for antifungal development, further in planta efficacy and safety studies are required before it can be considered for practical biocontrol.

## 1. Introduction

Wheat root and crown rot, primarily caused by *F. pseudograminearum*, *F. graminearum*, and *B. sorokiniana*, are among the most destructive soil-borne diseases of wheat worldwide [1,2]. These pathogens not only reduce grain yield and quality but also produce mycotoxins that pose risks to food safety [3,4]. Current disease management strategies rely heavily on chemical fungicides; however, concerns over fungicide resistance, environmental pollution, and public health have driven the search for safer and more sustainable alternatives, such as microbial biocontrol agents and their bioactive metabolites [5,6].

Endophytic microorganisms, including bacteria and fungi, live inside healthy plant tissues for at least a portion of their life cycle, typically causing no visible disease symptoms [7]. As a result of prolonged co-evolution with their host species, these microbes frequently produce bioactive secondary metabolites whose structures may match or resemble those synthesized by the plants themselves [8]. Within this group, fungi have drawn special research interest because they can form close symbiotic associations with their plant hosts. Genetic evidence supports molecular crosstalk between plants and arbuscular mycorrhizal fungi (AMF) [9], while Ordovician fossil records revealing fungal hyphae and spores morphologically indistinguishable from modern AMF suggest that such endophytic associations may have played a pivotal role during the early colonization of land by plants [10]. In the context of biological control, endophytic fungi suppress phytopathogens through multiple mechanisms, including niche competition, disruption of pathogen cell membrane integrity, and degradation of cell wall components, thereby inhibiting hyphal growth and spread [5,11]. Moreover, endophytic fungi are capable of producing structurally diverse and novel secondary metabolites, which not only represent adaptations to the host microenvironment but also serve as chemical weapons against competing organisms [6]. Consequently, endophytic fungi are considered promising sources of new pharmaceutical leads and potential candidates for agricultural biocontrol strategies.

Recently, during our screening of endophytic fungi isolated from *H. diffusa* collected in Zhumadian City, Henan Province, China, a *Fusarium* strain Y2 was identified, which exhibited significant antifungal activity against major wheat pathogens. *Fusarium* species are filamentous fungi found widely in nature. Many members of this genus are well-known plant pathogens that can produce various mycotoxins (e.g., zearalenone, moniliformin, and fumonisins), which contribute significantly to rot and wilt diseases in crops [3,4]. Nevertheless, genus *Fusarium* is also renowned for producing structurally unique secondary metabolites, including alkaloids, terpenoids, quinones, and polyketides—with diverse bioactivities such as antimicrobial and insecticidal, antitumor, antiviral, and antioxidant properties [12,13,14,15,16].

One such metabolite is equisetin, a tetramic acid derivative featuring a unique spirocyclic scaffold that was first isolated from *Fusarium equiseti* NRRL 5537 [17,18]. This compound has attracted considerable attention due to its diverse biological activities, including antibacterial effects against Gram-positive bacteria [17], inhibition of HIV-1 integrase [19], and anti-obesity activity through targeting 11 β-hydroxysteroid dehydrogenase type 1 [20]. More recently, equisetin has been reported to exhibit antifungal activity against *Botrytis cinerea* and *Sclerotinia sclerotiorum* [21,22]. However, its activity against major wheat root and crown rot pathogens, particularly *F. pseudograminearum* and *B. sorokiniana*, has not been previously explored. Furthermore, the transcriptional response of phytopathogenic fungi to equisetin treatment remains largely unknown.

In this study, we characterized the endophytic fungal strain Y2 isolated from *H. diffusa*, which displayed pronounced antifungal activity against three wheat pathogens: *F. graminearum*, *F. pseudograminearum*, and *B. sorokiniana*. Comprehensive morphological and molecular phylogenetic analyses were conducted to clarify its taxonomic identity, and pathogenicity assessment confirmed that strain Y2 is non-pathogenic to wheat, supporting its biosafety. Equisetin was successfully identified as the principal bioactive constituent from the extract of PDA fermentation of Y2. The inhibitory effects of equisetin against the aforementioned pathogens were confirmed, and transcriptomic analysis combined with qRT-PCR validation was performed to investigate the transcriptional response of *F. pseudograminearum* to equisetin treatment. These findings highlight equisetin as a promising lead compound for antifungal development and provide a foundation for future research on its potential application in cereal crop protection.

## 2. Materials and Methods

### 2.1. Materials

The endophytic fungal strain Y2, isolated from the leaves of healthy *H. diffusa*, along with the pathogenic strains *F. graminearum*, *F. pseudograminearum*, and *B. sorokiniana*, was preserved at the Institute of Mycology, College of Plant Protection, Henan Agricultural University, China.

### 2.2. Methods

#### 2.2.1. Antagonistic Activity of Endophytic Strain Y2 Against Three Major Wheat Root and Stem Pathogens

The antagonistic activity of strain Y2 against three wheat root and stem pathogens (*F. pseudograminearum*, *F. graminearum*, and *B. sorokiniana*) was evaluated using a dual-culture plate assay [23]. Strains Y2, and the pathogens were initially cultured on PDA plates for 3 days. Agar disks (5 mm diameter) containing mycelia of each target pathogen were cut from actively growing cultures and transferred to the center of fresh PDA plates. Subsequently, four 5-mm mycelial plugs of strain Y2 were positioned equidistantly around the central pathogen plug at a distance of 30 mm from its edge using sterile forceps. Control plates received sterile PDA plugs instead of Y2 inoculum at the same positions. Each treatment was performed in triplicate to ensure data reliability. All inoculated plates were incubated in the dark at 25 °C for 5 days. After inoculation, plates were incubated in darkness at 25 °C for five days, after which pathogen colony diameters were measured. The percentage inhibition of mycelial growth was determined using the formula: (Cd − Td) × 100%/(Cd − 0.5). In this equation, Cd and Td denote the colony diameters (in cm) measured for the control and treated groups, respectively, while 0.5 cm represents the diameter of the original mycelial plug used for inoculation. The experiment was performed in three independent biological replicates. Data are presented as mean ± standard deviation (SD). Statistical significance was analyzed using one-way ANOVA followed by Tukey‘s post-hoc test (GraphPad Prism 10, GraphPad Software, San Diego, CA, USA), with *p* < 0.05 considered statistically significant.

#### 2.2.2. Pathogenicity Assessment of Strain Y2 on Wheat Plants

To evaluate the potential phytopathogenicity of strain Y2 on wheat, a mycelial plug inoculation assay was performed on the stem bases of 45-day-old wheat plants (cv. Bainong 202). The stem bases were surface-sterilized with 75% ethanol for 3 min, rinsed three times with sterile distilled water, and air-dried. Mycelial plugs (5 mm diameter) of strain Y2 were cut from the actively growing margins of colonies on PDA using a sterile cork borer and attached to the stem bases using sterile forceps. Sterile PDA plugs served as the negative control, and *F. pseudograminearum* ZM20240321 was used as the pathogenic positive control. The inoculated plants were covered with plastic film for 24 h to maintain high humidity and incubated at 25 °C under a 12 h light/dark photoperiod. The inoculation plugs were removed after 24 h, and lesion length was measured and photographed at 5 days post-inoculation (dpi). The experiment was performed independently three times, with 10 replicates per treatment. Statistical analysis was performed using GraphPad Prism 10 software by one-way ANOVA followed by Tukey’s post-hoc test (*p* < 0.05). Data are presented as mean ± SD.

#### 2.2.3. Morphological Characterization of Endophytic Strain Y2

Strain Y2 was aseptically transferred to the center of PDA plates and then allowed to grow at 25 °C for one week. After this period, the macroscopic features of the colonies—such as their morphology, pigmentation (on both the front and reverse sides), texture, and overall size—were documented. Colony diameters were determined using the cross-measurement method. For microscopic observation, spores were picked up using a sterile needle, suspended in a droplet of distilled water on a glass slide, and then gently covered with a coverslip. The shape and dimensions of the spores were examined and recorded under a light microscope.

#### 2.2.4. Molecular Identification of Endophytic Strain Y2

To further identify strain Y2 at the molecular level, its genomic DNA was first extracted using the cetyltrimethylammonium bromide (CTAB) protocol. The purified DNA was then used as a PCR template with two primer sets: EF1/EF2 [24], which target the translation elongation factor 1-α (*TEF-1α*) gene, and 5f2/7cr [25], which target the second-largest subunit of RNA polymerase II (*RPB2*) gene. The sequences of all primers are provided in Table 1. Each PCR reaction was prepared in a total volume of 20 μL, containing 1 μL of template DNA, 1 μL of each primer (10 μmol·L^−1^), 7 μL of 2× Taq PCR Master Mix, and 10 μL of sterile double-distilled water. The thermocycling conditions were set according to previously established protocols [24,25]. The resulting PCR products were resolved by electrophoresis at 120 V for 30 min, then visualized under a gel imaging system and photographed to confirm the correct amplicon sizes. After purification, the products were sent to a commercial sequencing provider for bidirectional sequencing. The obtained *TEF-1α* and *RPB2* sequences from strain Y2 were compared against homologous sequences in the NCBI database using the BLAST algorithm (NCBI BLAST+ v2.13.0, https://blast.ncbi.nlm.nih.gov).

For phylogenetic analysis, reference sequences were selected from previously published studies on the *F. incarnatum-equiseti* species complex [26,27], covering major phylogenetic lineages within this complex. The dataset included 24 reference *Fusarium* strains, with *Stemphylium vesicarium* serving as the outgroup. The taxonomic designations and GenBank accession numbers for these reference strains are summarized in Table 2. Multiple sequence alignment was carried out with ClustalW. The concatenated alignment length was 2583 bp, including 695 parsimony-informative sites. The best-fit nucleotide substitution model, GTR + G (General Time Reversible with Gamma distribution), was selected based on the Akaike Information Criterion (AIC) using jModelTest v2.1.10. The dataset was partitioned by gene region (*TEF-1α* and *RPB2*) for phylogenetic reconstruction. A multi-gene dataset was then assembled and analyzed using PhyloSuite v1.2.1, and a maximum-likelihood (ML) phylogenetic tree was built with IQ-TREE v1.6.12, with nodal support assessed using 1000 bootstrap replicates, to determine the phylogenetic placement of strain Y2 in relation to its close relatives. While type strains were not available for all reference sequences, the selected reference strains represent well-characterized isolates from published taxonomic studies, which is a widely accepted practice in FIESC systematics given the current complexity of this species complex [25,26].

#### 2.2.5. Fermentation, Extraction and Isolation, and Structure Determination

The fungal strain was cultivated on PDA medium for five days at 28 °C. Following fermentation, a total of 100 PDA culture disks were dried at 45 °C for two days, after which they were extracted with ethyl acetate (EtOAc) at ambient temperature. The resulting EtOAc extract was then evaporated under reduced pressure, yielding 1.88 g of crude extract. This crude material was analyzed by HPLC (Agilent 1100, Santa Clara, CA, USA) using an Agilent ZORBAX SB-C8 column (5 μm, 150 × 4.6 mm, CA, USA) equipped with a diode-array detector (DAD). The elution followed a gradient program: 10–100% acetonitrile in water from 0 to 30 min, followed by 100% acetonitrile from 30 to 35 min. For purification, the crude extract was subjected to semi-preparative HPLC (Waters 2695, Milford, MA, USA) fitted with an Agilent ZORBAX SB-C18 column (5 μm, 250 × 9.4 mm, CA, USA). To elucidate the structure of compound **1**, NMR spectra were acquired in CDCl_3_ on a Bruker Avance III 600 MHz spectrometer (Bruker, Berlin, Germany). HR-ESI-MS of compound **1** was obtained on a Thermo Scientific Q Exactive spectrometer (Waltham, MA, USA).

#### 2.2.6. Determination of the Minimum Inhibitory Concentration (MIC) of Equisetin and the Crude Extract

To determine the minimum inhibitory concentration (MIC) of equisetin and the crude extract, a microbroth dilution assay was performed using 96-well microtiter plates against *B. sorokiniana*, *F. pseudograminearum*, and *F. graminearum*. Spore suspensions were prepared by culturing *B. sorokiniana* on PDA and both *Fusarium* species on Carboxymethyl Cellulose (CMC) agar at 25 °C for 7 days. Spores were harvested with sterile distilled water containing 0.05% Tween-80, filtered through sterile lens paper to remove mycelial fragments, and adjusted to a concentration of 1 × 10^5^ spores/mL in Potato Dextrose Broth (PDB). The crude extract was obtained from 7-day-old PDA cultures of *F. incarnatum* Y2 by extraction with ethyl acetate, followed by filtration and vacuum concentration to dryness. For the assay, two-fold serial dilutions of equisetin and the redissolved crude extract were performed in PDB supplemented with 0.5% DMSO and 0.01% Tween-80. Both test substances were diluted to generate the same concentration gradient. Specifically, 100 μL of each diluted solution (at twice the final desired concentration) was dispensed into the wells, followed by the addition of 100 μL of the standardized spore suspension. This resulted in final test substance concentrations of 256, 128, 64, 32, 16, 8, and 4 μg/mL and a final inoculum density of approximately 5 × 10^4^ spores/mL in a total volume of 200 μL. The experiment included a growth control (spores with solvent), a positive control [carbendazim (50% wettable powder), which was first dissolved in dilute hydrochloric acid and then diluted with sterile distilled water to a final active concentration of 8 μg/mL], and a sterility control (medium only). Plates were sealed and incubated at 25 °C for 48 h. The MIC assay was performed in three independent biological replicates. MIC endpoints were determined visually as the lowest concentration showing no visible fungal growth. Solvent controls containing 0.5% DMSO and 0.01% Tween-80 (equivalent to the highest concentration in test wells) were included in all treatments and showed no inhibitory effects.

#### 2.2.7. Transcriptome Analysis

The strain *F. pseudograminearum* ZM20240321 was taken from a 4 °C refrigerator and inoculated onto PDA plates, then incubated in a biochemical incubator at 25 °C in the dark for 3 days. Five mycelial plugs (5 mm in diameter) were taken from the colony margin using a 5 mm cork borer and inoculated into 250 mL Erlenmeyer flasks containing 150 mL of PDB liquid medium. A total of six flasks were prepared, with three serving as the control group and the other three as the equisetin-treated group. The flasks were placed on a rotary shaker at 25 °C and 180 rpm. After 36 h of cultivation, 240 μL of equisetin (10 mg/mL) was added to each flask in the treatment group, while 240 μL of DMSO was added to each control flask. Cultivation continued for another 12 h. Mycelia were then harvested by filtration through two layers of sterilized filter cloth and washed thoroughly with sterile water. The washed mycelia were immediately transferred into 2 mL centrifuge tubes, frozen rapidly in liquid nitrogen, and sent to Shanghai Personal Biotechnology Co., Ltd. for RNA extraction and subsequent RNA sequencing analysis. RNA-seq was performed on an Illumina NovaSeq X PLUS platform (Illumina, San Diego, CA, USA) using a stranded RNA-seq library with 150 bp paired-end reads. After quality filtering, approximately 40.6–46.1 million clean reads were obtained per sample, with Q30 values exceeding 95.23% and GC contents ranging from 52.59% to 53.23%. The clean reads were mapped to the *F. pseudograminearum* Class2-1C reference genome (GenBank assembly GCA_016952305.1) using HISAT2 v2.1.0, with an average mapping rate of >85%. Gene expression levels were quantified using featureCounts v2.0.1 and normalized as FPKM (fragments per kilobase of exon per million mapped fragments). Differential expression analysis was performed using DESeq2 v1.30.1. Genes were considered differentially expressed if they met strict criteria of a false discovery rate (FDR) below 0.05 and an absolute log_2_ fold change of at least 1. Functional enrichment analyses for gene ontology (GO) terms and Kyoto Encyclopedia of Genes and Genomes (KEGG) pathways were then carried out with the ClusterProfiler software package (v4.4.4) with the *F. pseudograminearum* genome as the background gene set. Enrichment significance was determined using a hypergeometric test, and *p*-values were adjusted for multiple hypothesis testing using the Benjamini–Hochberg false discovery rate (FDR) method, with significance defined as adjusted *p*-value < 0.05. The raw RNA-seq data have been deposited in the NCBI Sequence Read Archive (SRA) under accession number PRJNA1483097.

#### 2.2.8. qRT-PCR Validation of Differentially Expressed Genes

To validate the RNA-seq results, seven representative differentially expressed genes were selected for qRT-PCR analysis based on their involvement in the most significantly enriched pathways: four upregulated genes from the ribosome pathway—*FpRPL2* (ribosomal protein L2), *FpRPL24* (ribosomal protein L24), *FpRPS0* (ribosomal protein S0), and *FpRPS27a* (ribosomal protein S27a)—and three downregulated genes from the nitrogen metabolism pathway—*FpCA2* (carbonic anhydrase 2), *FpGLN1* (glutamate–ammonia ligase), and *FpGDH2* (glutamate dehydrogenase). Total RNA was isolated using Trizol reagent (Aidlab Biotech Co., Ltd., Beijing, China) following the manufacturer‘s protocol. Purified RNA was reverse-transcribed into cDNA using the SuperScript III First-Strand Synthesis System Kit (Vazyme Biotech Co., Ltd., Nanjing, China), and the resulting cDNA served as templates for qRT-PCR with ChamQ Universal SYBR qPCR Master Mix (Vazyme Biotech Co., Ltd., Nanjing, China). The *FpTEF1α* gene was used as the internal reference for normalization of gene expression [28]. Relative transcript abundance was calculated via the 2^−ΔΔCt^ method. All primer sequences are listed in attached Table S1.

## 3. Results

### 3.1. Antagonistic Activity of Endophytic Strain Y2 Against Three Major Pathogens of Wheat Roots and Crowns

**Figure 1:**
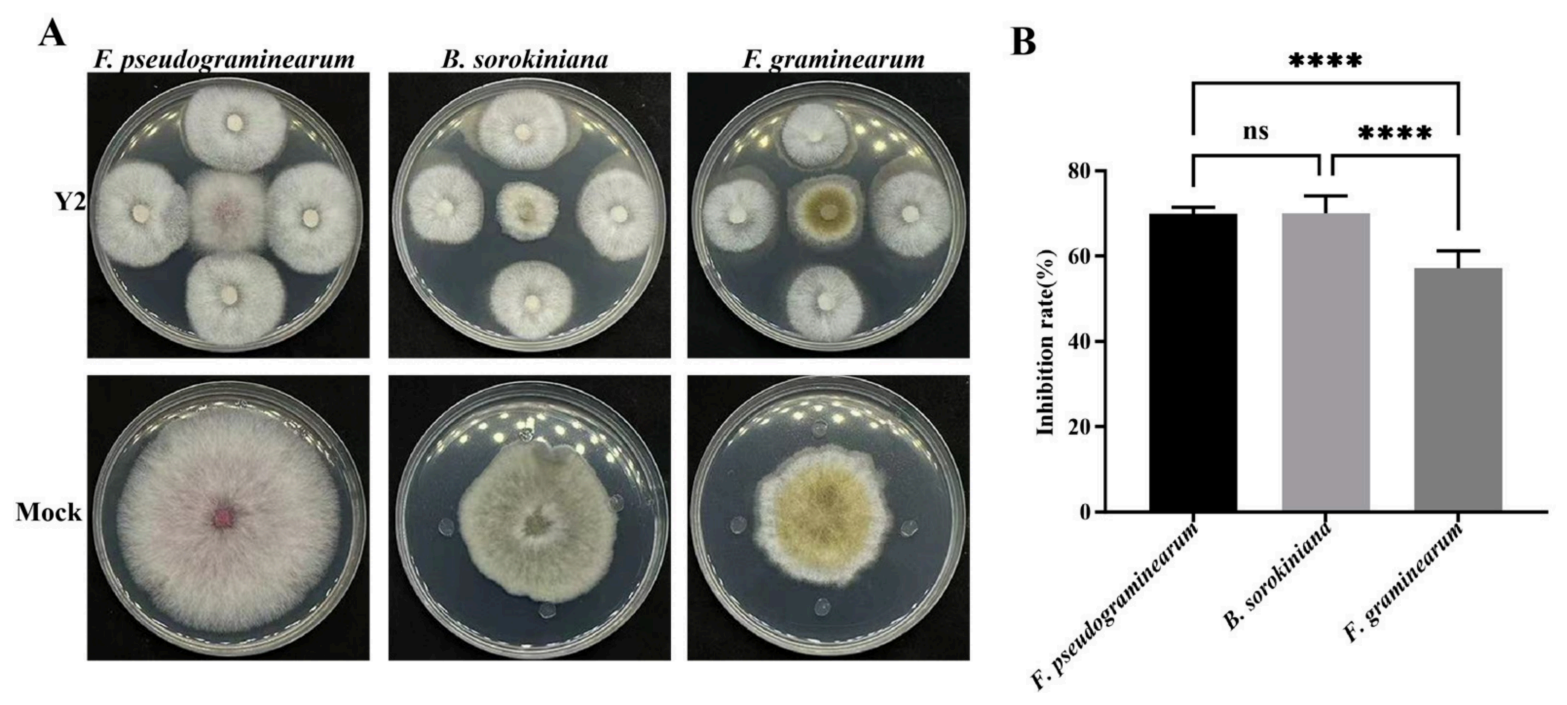
Inhibitory effects and inhibition rates of endophytic fungus Y2 against three major pathogens of wheat roots and crowns. (**A**) Dual-culture assays showing the antagonistic activity of strain Y2 against the three pathogens on PDA plates. (**B**) Quantitative analysis of the inhibition rates (%) of strain Y2 against the tested pathogens. Data are presented as mean ± standard deviation (SD) of three independent experiments. Statistical significance was analyzed using one-way ANOVA followed by Tukey’s post-hoc test. Bars with different annotations indicate significant differences: ns, not significant (*p* > 0.05); ****, *p* < 0.0001.

The antagonistic activity of endophytic strain Y2 against three major pathogens affecting wheat roots and crowns was evaluated using a five-point dual-culture assay on PDA plates. Strain Y2 exhibited significant inhibitory effects against all three tested pathogens (Figure 1A). The inhibition rates against *F. pseudograminearum* (69.63%) and *B. sorokiniana* (70.01%) were comparable, with no significant difference between them (*p* > 0.05). In contrast, the inhibition rate against *F. graminearum* (57.22%) was significantly lower than that against the other two pathogens (*p* < 0.0001) (Figure 1B). These results indicate that while strain Y2 displays a broad-spectrum antagonistic activity against major wheat crown and root rot pathogens, its efficacy varies among species, with significantly stronger inhibition against *F. pseudograminearum* and *B. sorokiniana* than against *F. graminearum*.

### 3.2. Pathogenicity of Strain Y2 on Wheat Plants

**Figure 2:**
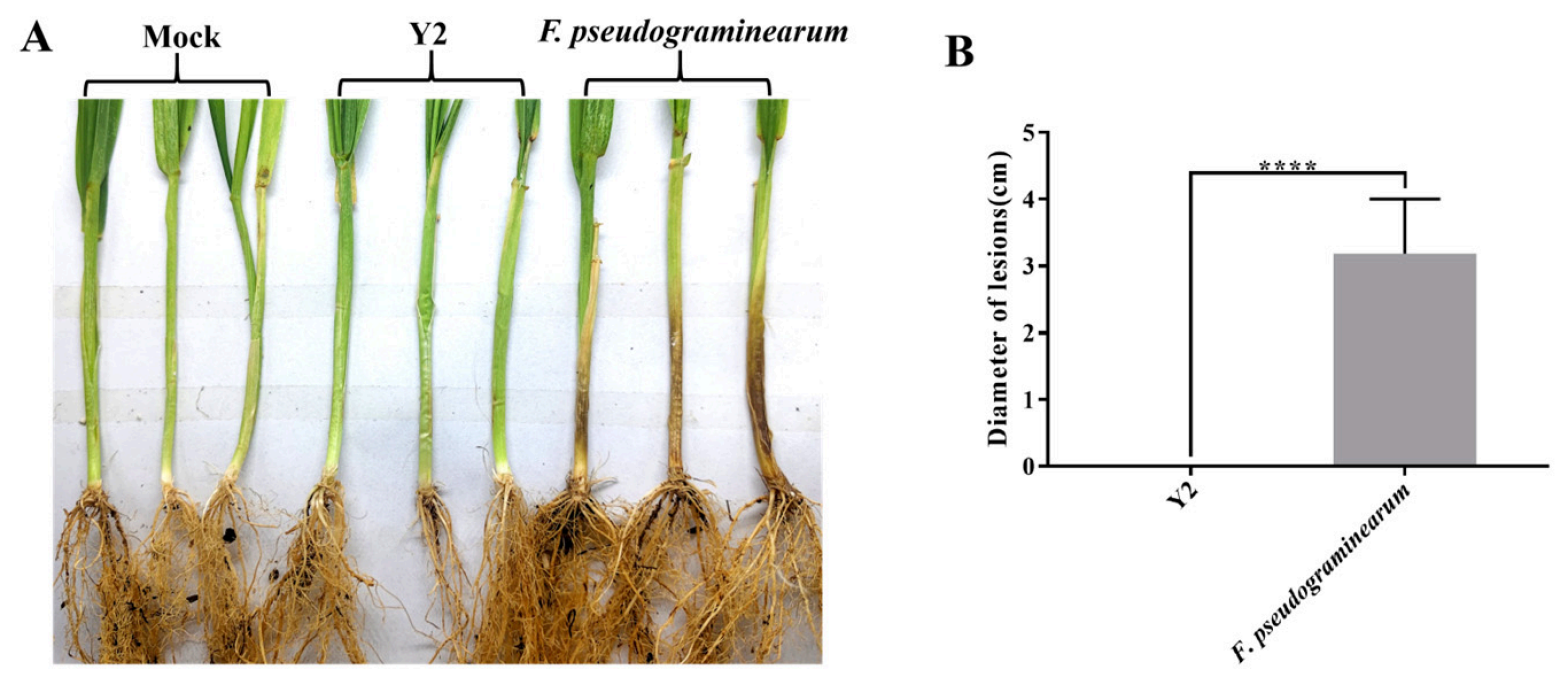
Pathogenicity assessment of endophytic fungus Y2 on wheat stem bases at 5 days post-inoculation. (**A**) Representative phenotypes of wheat stem bases inoculated with sterile PDA (negative control), endophytic fungus Y2, and *F. pseudograminearum* ZM20240321 (positive control). (**B**) Quantitative analysis of lesion length. Data are presented as mean ± standard deviation (SD) of three independent experiments. **** indicates significant difference compared to the negative control at *p* < 0.0001 (one-way ANOVA with Tukey‘s post-hoc test).

To evaluate the biosafety of strain Y2, a wheat stem base inoculation assay was performed using 45-day-old wheat plants. At 5 days post-inoculation, wheat plants inoculated with *F. pseudograminearum* ZM20240321 (positive control) exhibited typical crown rot symptoms, characterized by extensive brown necrotic lesions at the stem base, with an average lesion length of 3.19 cm. In contrast, seedlings inoculated with strain Y2 showed no visible disease symptoms (lesion length = 0 cm), identical to the negative control (sterile PDA) which also showed no symptoms (Figure 2). These results clearly demonstrate that strain Y2 is non-pathogenic to wheat under the tested conditions, supporting its safety as a candidate strain for further biocontrol-related investigations.

### 3.3. Morphological Identification of Strain Y2

**Figure 3:**
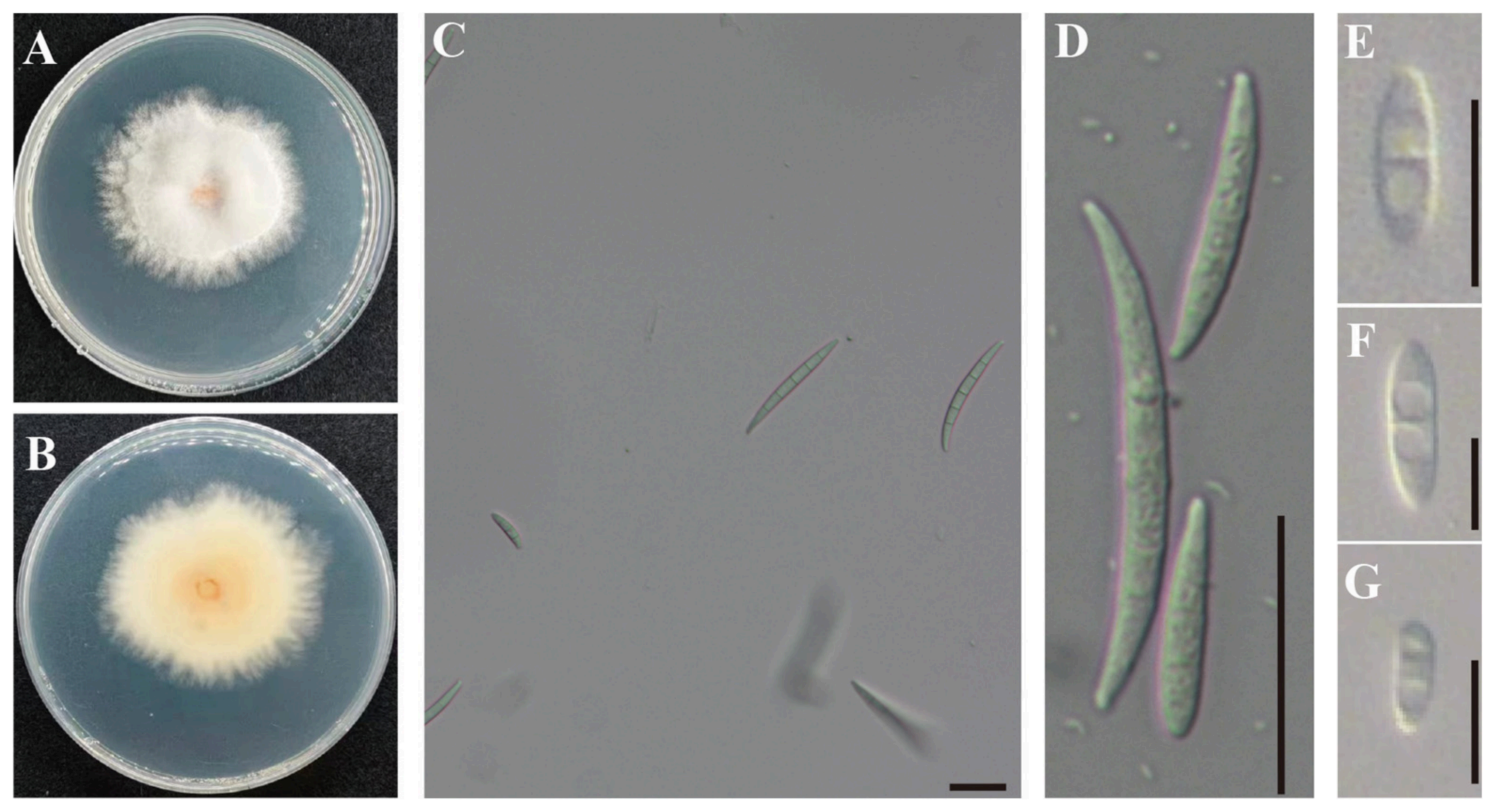
Morphological characteristics of strain Y-2. (**A**) Colony morphology on the obverse side on PDA medium. (**B**) Colony morphology on the reverse side on PDA medium. (**C**,**D**) Macroconidia; scale bar = 20 μm. (**E**–**G**) Microconidia; scale bar = 10 μm.

After 7 days of incubation in the dark at 25 °C on PDA, colonies of strain Y2 reached a diameter of 5.4 ± 0.35 cm. The colonies were circular, characterized by dense white aerial mycelium radiating from the center, with sparser growth at the margins. The colony reverse transitioned from an initial white to light orange-yellow; by day 7, the central region exhibited intense pigmentation (light orange-yellow), while the margins remained pale, approaching white (Figure 3A,B). Microscopic examination revealed two distinct conidial types. Macroconidia were falcate (sickle-shaped), slender, and tapered at the apical cell, with a distinct foot cell at the basal end. They were predominantly 3 to 5 septate, measuring 20.46–47.99 μm × 3.02–5.23 μm (Figure 3C,D). Microconidia were oval to ovoid, mostly 1-septate, measuring 4.50–17.03 μm × 1.50–3.21 μm (Figure 3E–G). Based on these characteristic morphological features, strain Y2 was preliminarily identified as a species of genus *Fusarium*.

### 3.4. Molecular Identification of Strain Y2

**Figure 4:**
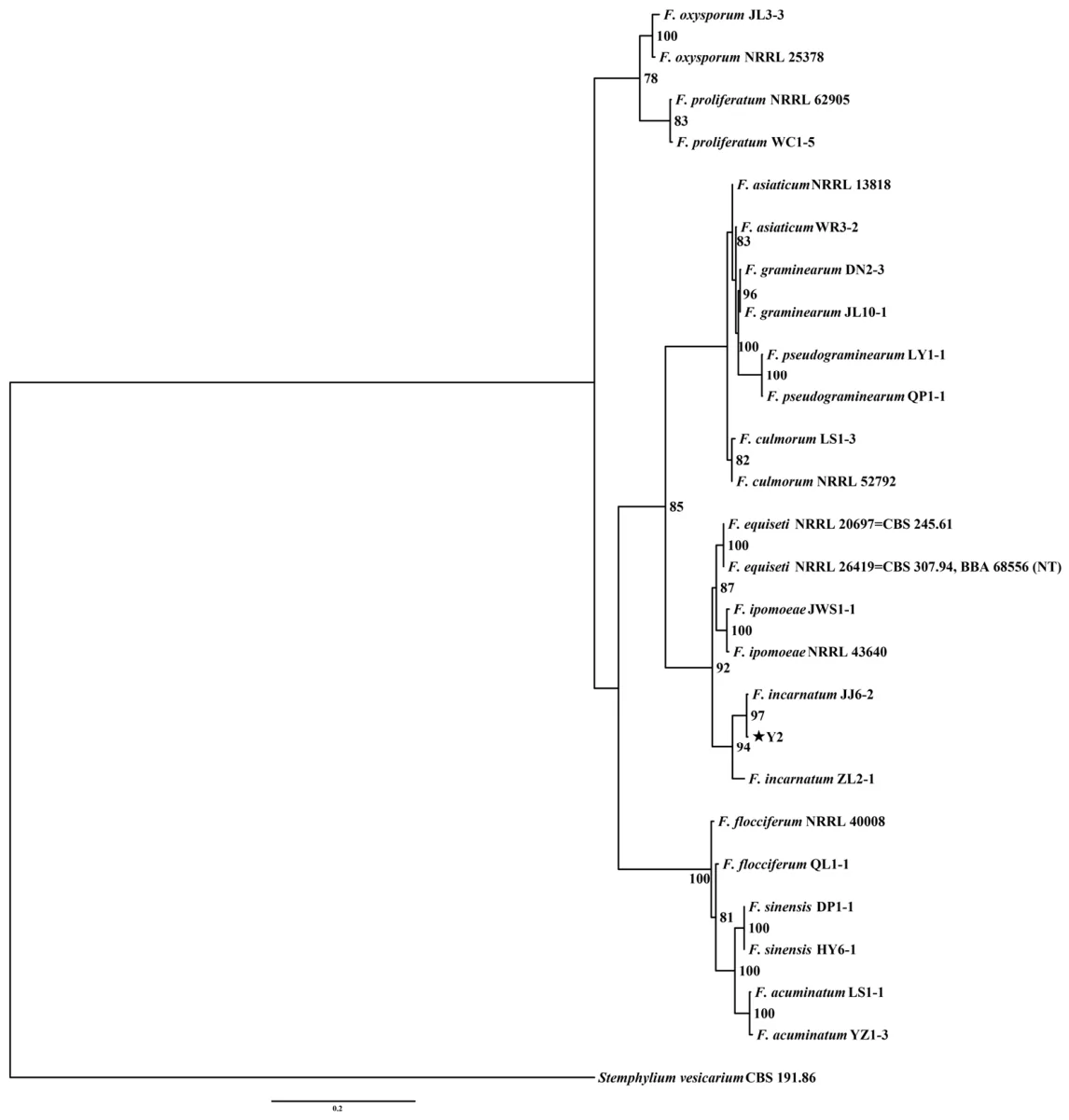
Maximum likelihood phylogenetic tree inferred from the combined *TEF1-α* and *RPB2* gene sequences of isolate Y2. Branch nodes are annotated with bootstrap support values calculated from 1000 resampling replicates. The scale bar corresponds to the estimated number of substitutions per sequence position. The star symbol (★) indicates the position of strain Y2.

To further elucidate the taxonomic status of endophyte Y2, genomic DNA was extracted, and the translation elongation factor 1-α (*TEF1-α*) and the second largest subunit of RNA polymerase II (*RPB2*) genes were amplified via PCR and sequenced. The obtained nucleotide sequences were deposited in GenBank under accession numbers PX964254 (*TEF1-α*) and PX964255 (*RPB2*). BLASTn analysis revealed that these sequences shared 99.14% to 99.69% identity with *TEF1-α* (OP105211.1) and *RPB2* (ON693005.1) sequences from *F. incarnatum* isolates. For phylogenetic analysis, a maximum likelihood (ML) tree was constructed based on the concatenated *TEF1-α* and *RPB2* sequences. The dataset included 24 reference *Fusarium* strains, with *S. vesicarium* serving as the outgroup. The resulting phylogeny showed that strain Y2 clustered closely with two other *F. incarnatum* isolates in a well-supported clade (Figure 4). Combining these molecular data with the aforementioned morphological characteristics, strain Y2 was identified as *F. incarnatum* or a closely related member of the *F. incarnatum–equiseti* species complex (FIESC).

### 3.5. Isolation and Structural Analysis of Bioactive Metabolites from F. incarnatum Y2

**Figure 5:**
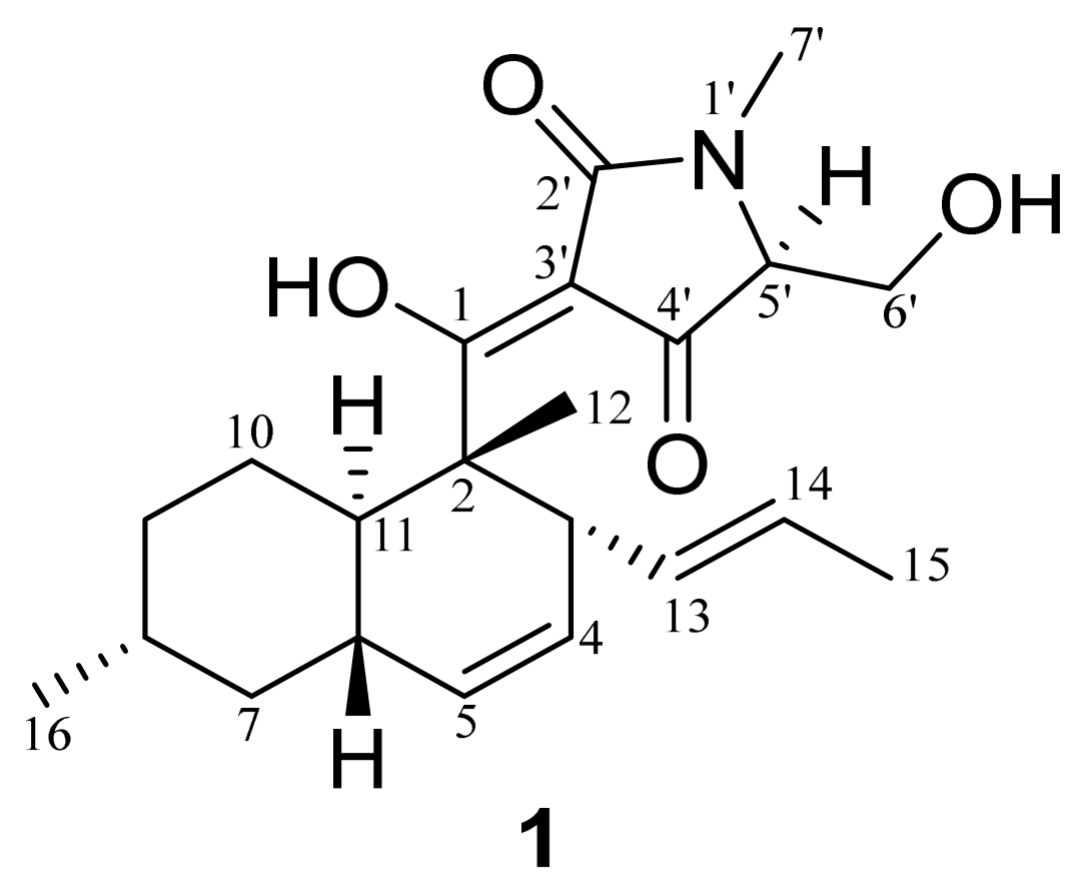
Chemical structure of **1**.

The extract was firstly subjected to HPLC analysis, and a main chromatographic peak was observed in the HPLC spectrum (attached Figure S1). The compound was separated by semi-preparative HPLC, eluting with acetonitrile–water (90:10, *v*/*v*) at a flow rate of 3 mL/min to obtain compound **1** (38.8 mg). The purity of compound **1** was confirmed by HPLC analysis (attached Figure S2) prior to NMR detection. The structure of **1** was identified as equisetin (Figure 5) by HR-ESI-MS ion peak at *m*/*z* 374.2324 [M+H]^+^ (calcd for C_22_H_32_NO_4_ 374.2326) (attached Figure S3) and by comparison of its 1D NMR spectra (attached Figures S4 and S5) with those in the literature [29]. The NMR data of **1** are listed as follows: ^1^H NMR (600 MHz, CDCl_3_) *δ*_H_: 5.40 (2H, m, H-4, H-5), 5.30–5.19 (2H, m, H-14, H-13), 4.02 (1H, dd, *J* = 11.6, 3.9 Hz, H-6′a), 3.89 (1H, m, H-6′b), 3.63 (1H, brs, H-5′), 3.49 (1H, s, H-3), 3.05 (3H, s, H-7′), 1.97 (1H, brs, H-10b), 1.85 (1H, overlap, H-6), 1.81 (1H, overlap, H-7b), 1.76 (1H, overlap, H-9b), 1.68 (1H, brs, H-11), 1.54 (3H, overlap, H-15), 1.50 (1H, overlap, H-8), 1.46 (3H, s, H-12), 1.13 (1H, overlap, H-9a), 1.06 (1H, overlap, H-10a), 0.92 (3H, d, *J* = 6.5 Hz, H-16), 0.89 (1H, overlap, H-7a); ^13^C NMR (150 MHz, CDCl_3_) *δ*_C_: 199.1 (C-4′), 190.5 (C-1), 177.1 (C-2′), 130.9 (C-5), 130.0 (C-13), 127.1 (C-4), 126.6 (C-14), 100.0 (C-3′), 66.8 (C-5′), 60.4 (C-6′), 48.8 (C-2), 45.0 (C-3), 42.2 (C-7), 39.9 (C-11), 38.6 (C-6), 35.7 (C-9), 33.5 (C-8), 28.3 (C-10), 27.3 (C-7′), 22.5 (C-16), 18.0 (C-15), 14.0 (C-12).

### 3.6. Antifungal Potency of the Crude Extract and Equisetin

The antifungal efficacy of equisetin and the crude extract was evaluated against three phytopathogenic fungi: *B. sorokiniana*, *F. pseudograminearum*, and *F. graminearum*. The data presented in Table 3 indicate that equisetin effectively suppressed the growth of all three fungal pathogens tested, although the degree of inhibition varied across species. The strongest effect was observed against *F. pseudograminearum*, for which the MIC was 16 μg/mL. In contrast, equisetin showed weaker activity against *B. sorokiniana* (MIC: 32 μg/mL) and *F. graminearum* (MIC: 64 μg/mL). In contrast, the crude extract containing equisetin displayed a broader but less potent inhibitory spectrum. It showed a uniform MIC value of 128 µg/mL against all three fungal species. For comparative reference, the commercial fungicide carbendazim exhibited consistent and potent activity against all three pathogens, with an MIC of 8 µg/mL for each. The MIC values reported in Table 3 were consistent across three independent replicates, with no variation observed between replicates. These results highlighted the potential of equisetin as a lead compound for further antifungal evaluation.

### 3.7. Differential Expression Profile and Global Transcriptomic Response

**Figure 6:**
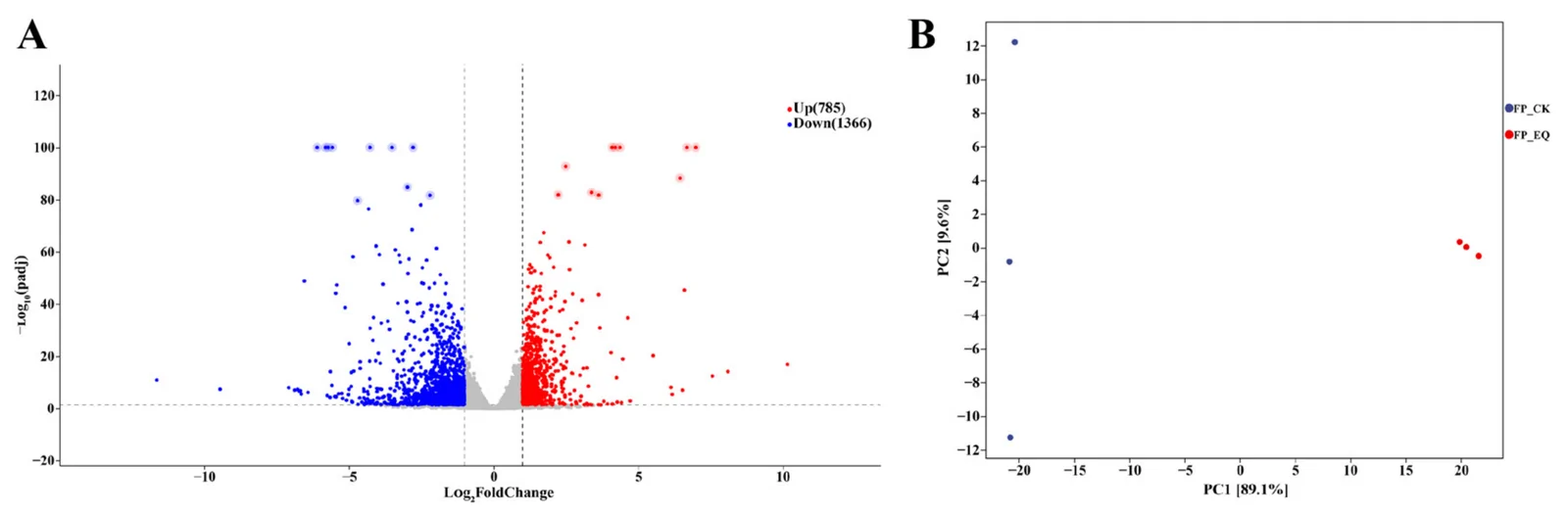
Transcriptomic profiling of *F. pseudograminearum* treated with equisetin. (**A**) Volcano plot of differentially expressed genes (DEGs). Using thresholds of |log_2_ fold change| ≥ 1 and FDR < 0.05, a total of 2151 DEGs were identified, including 785 upregulated genes (red) and 1366 downregulated genes (blue). (**B**) Principal component analysis (PCA) of transcriptomes. The control and equisetin-treated groups are clearly separated along the first principal component (PC1, explaining 89.1% of the variance), while the second principal component (PC2, explaining 9.6% of the variance) shows no clear separation within groups.

To investigate the transcriptional regulation of *F. pseudograminearum* ZM20240321 by 16 μg/mL equisetin, RNA-seq analysis was performed. Using |log_2_FC| ≥ 1 and FDR < 0.05 as thresholds, a total of 2151 DEGs were identified, including 785 upregulated and 1366 downregulated genes (Figure 6A). The number of downregulated genes is approximately 1.74 times that of upregulated genes. The volcano plot clearly showed that downregulated genes dominate the overall distribution, indicating that equisetin treatment exerts a stronger suppressive than activating effect on the transcriptome. PCA results showed that PC1 explained 89.1% of the total variance and PC2 explained 9.6% (Figure 6B). The control and equisetin-treated groups were completely separated along the PC1 axis, with tight clustering within each group. This indicated that the treatment effect was the major driver of transcriptomic changes and that experimental reproducibility was good. Although downregulated genes significantly outnumber upregulated ones, a key “polarization phenomenon” was observed: in the subsequent GO enrichment analysis (103 significant terms; see attached Table S2), almost all terms were dominated by upregulated genes, with some terms (e.g., “cytosolic ribosome”, “translation”) showing 100% upregulation. This seemingly paradoxical result suggested that equisetin did not act through global transcriptional inhibition but rather appeared to preferentially activate ribosome and translation-related pathways while causing widespread downregulation of non-core functional genes.

### 3.8. Functional Enrichment Analysis

**Figure 7:**
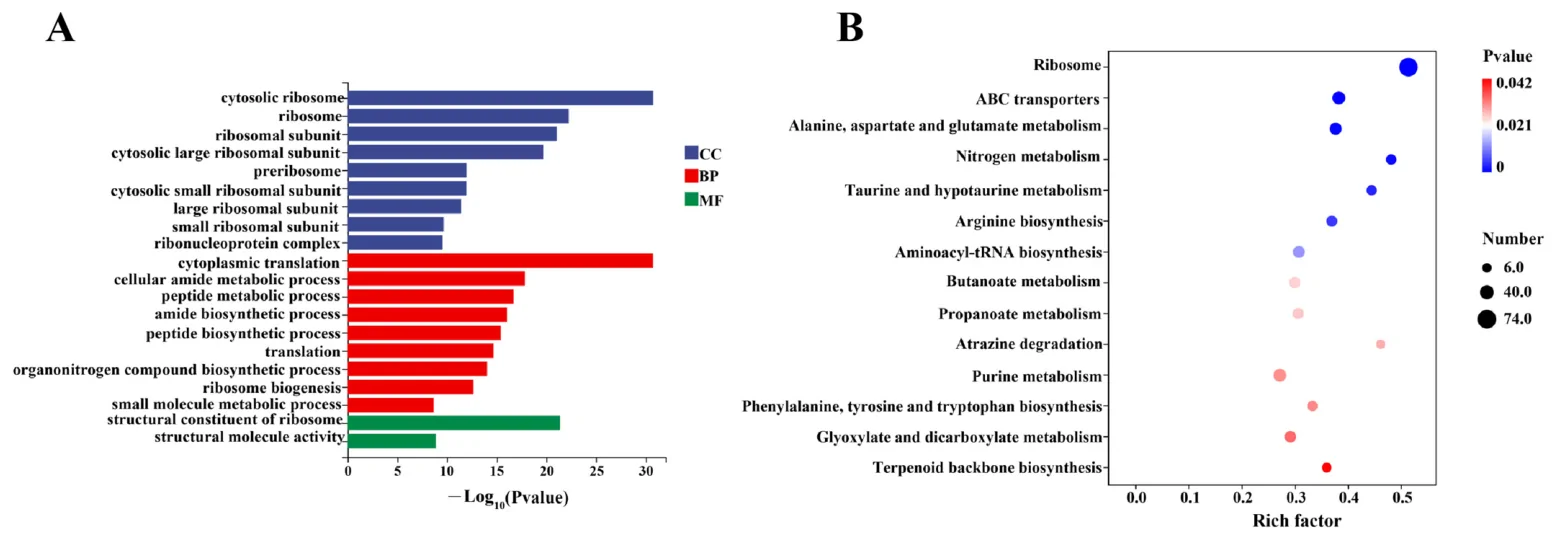
GO and KEGG enrichment analysis of differentially expressed genes in *F. pseudograminearum* treated with equisetin. (**A**) GO enrichment analysis. (**B**) KEGG pathway enrichment analysis.

GO functional enrichment analysis yielded 103 significant terms (adjust *p*-value < 0.05), covering cellular component (CC), biological process (BP), and molecular function (MF) (attached Table S2). These terms were highly concentrated on ribosome structure, translation, and ribosome biogenesis, and all were dominated by upregulated genes (Figure 7A). In terms of cellular component, the most significant terms included “cytosolic ribosome” (GO:0022626, 69 up/0 down), “ribosome” (GO:0005840, 83 up/0 down), “ribosomal subunit” (GO:0044391, 73 up/0 down), “cytosolic large ribosomal subunit” (GO:0022625, 36 up/0 down), and “cytosolic small ribosomal subunit” (GO:0022627, 29 up/0 down), indicating that equisetin strongly induced global expression of ribosomal protein genes. Regarding biological processes, the main terms centered on translation and its upstream events, such as “cytoplasmic translation” (GO:0002181, 107 up/0 down), “peptide biosynthetic process” (GO:0043043, 130 up/1 down), and “amide biosynthetic process” (GO:0043604, 141 up/6 down). Additionally, “ribosome biogenesis” (GO:0042254, 105 up/2 down), “rRNA processing” (GO:0006364, 69 up/1 down), and “ribosomal large subunit assembly” (GO:0000027, 15 up/1 down) were also highly significant, demonstrating that equisetin not only activated translation itself but also promoted de novo ribosome assembly and maturation. In terms of molecular function, core terms included “structural constituent of ribosome” (GO:0003735, 69 up/0 down), “structural molecule activity” (GO:0005198, 71 up/2 down), “aminoacyl-tRNA ligase activity” (GO:0004812, 19 up/0 down), and “unfolded protein binding” (GO:0051082, 23 up/2 down), further supporting the global activation of the translational machinery and suggesting possible protein folding stress. Notably, among these 103 significant GO terms, none was dominated by downregulated genes; even in terms containing a small number of downregulated genes, such as “small molecule metabolic process” (123 up/64 down), the overall trend remained predominantly upward.

KEGG pathway enrichment analysis identified four significantly enriched pathways (adjust *p*-value < 0.05) (Figure 7B). Among them, the ribosome pathway (map03010) was the most significant (adjust *p*-value = 4.01 × 10^−16^), containing a large number of differentially expressed genes that were predominantly upregulated. The ABC transporters pathway (map02010) contained differentially expressed genes with similar numbers of up- and downregulated genes. The alanine, aspartate and glutamate metabolism pathway (map00250) was also significantly enriched. The nitrogen metabolism pathway (map00910) was the only significantly enriched pathway dominated by downregulated genes. These results indicated that equisetin treatment most significantly affected ribosome function, while also moderately altered pathways related to membrane transport, amino acid metabolism, and nitrogen metabolism.

### 3.9. qRT-PCR Validation of Key Differentially Expressed Genes

**Figure 8:**
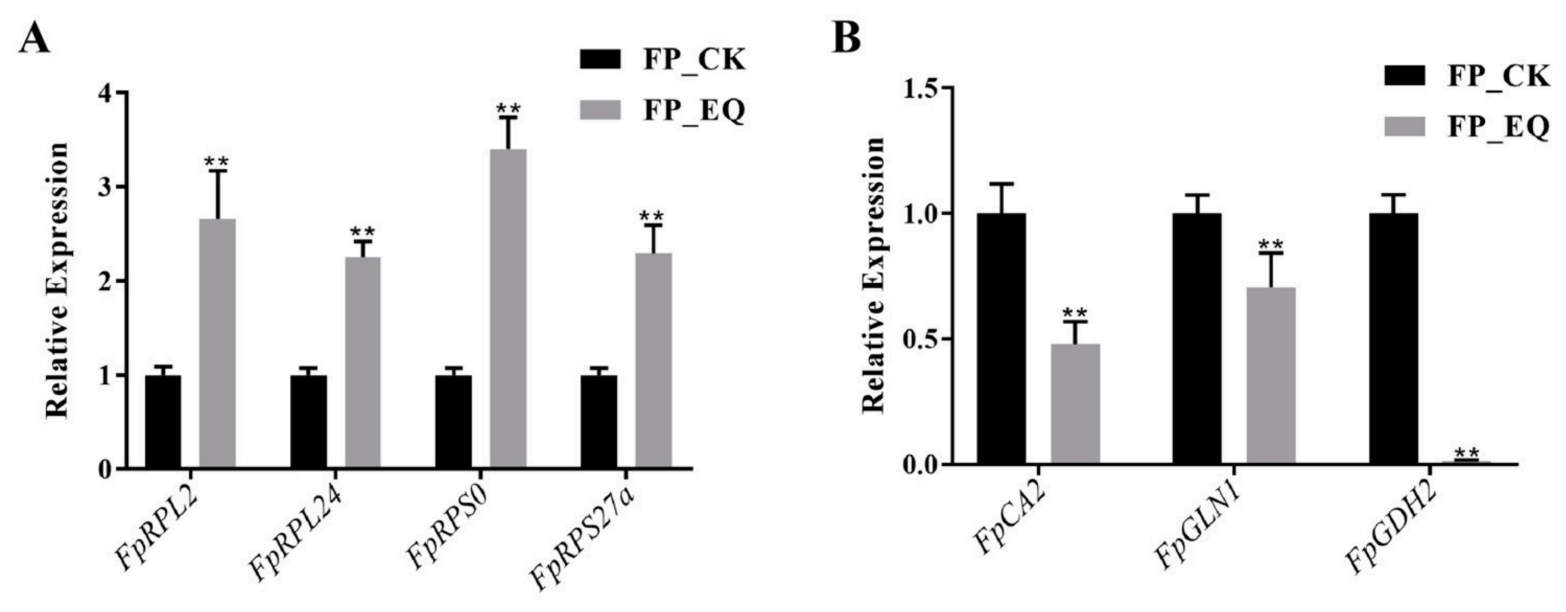
qRT-PCR validation of RNA-seq data for selected differentially expressed genes. Relative expression levels of seven representative genes in *F. pseudograminearum* under control (FP_CK) and equisetin-treated (FP_EQ) conditions. (**A**) Four upregulated ribosomal protein genes (*FpRPL2*, *FpRPL24*, *FpRPS0*, and *FpRPS27a*). (**B**) Three downregulated nitrogen metabolism genes (*FpCA2*, *FpGLN1*, and *FpGDH2*). Asterisks indicate significant differences (Student’s *t*-test, ** *p* < 0.01).

To validate the reliability of the RNA-seq data, seven representative differentially expressed genes were selected for qRT-PCR analysis based on their involvement in the most significantly enriched pathways: four upregulated genes from the ribosome pathway (*FpRPL2*, *FpRPL24*, *FpRPS0*, and *FpRPS27a*) and three downregulated genes from the nitrogen metabolism pathway (*FpCA2*, *FpGLN1*, and *FpGDH2*). The qRT-PCR results confirmed the expression trends observed in the RNA-seq data for all seven selected genes (Figure 8). For the ribosome pathway, the four ribosomal protein genes showed consistent upregulation: *FpRPL2* (RNA-seq log_2_FC = 1.148; qRT-PCR fold change = 2.66), *FpRPL24* (log_2_FC = 1.2123; qRT-PCR = 2.25), *FpRPS0* (log_2_FC = 1.8774; qRT-PCR = 3.40), and *FpRPS27a* (log_2_FC = 1.2595; qRT-PCR = 2.29). For the nitrogen metabolism pathway, the three genes exhibited consistent downregulation: *FpCA2* (log_2_FC = −1.029; qRT-PCR = 0.47), *FpGLN1* (log_2_FC = −1.194; qRT-PCR = 0.70), and *FpGDH2* (log_2_FC = −6.532; qRT-PCR = 0.01). The concordant directional changes across all seven genes substantiate the reliability of the transcriptomic analysis.

## 4. Discussion

Endophytic fungi are recognized as rich sources of novel natural products and have become a crucial resource bank for the discovery of agrochemicals [8,30]. Although *Fusarium* species include many notorious plant pathogens, they are also prolific producers of structurally novel and biologically diverse secondary metabolites [31]. The significant antagonistic activity of *F. incarnatum* Y2 against tested wheat pathogens aligned with previous reports on the antimicrobial potential of endophytic *Fusarium* species [13,15]. This study further enriched our understanding of the ecological function of *F. incarnatum* as an endophyte and represented the first report of its colonization in *H. diffusa*.

Accurate identification of *Fusarium* species is fundamental to related research. Due to high morphological similarity and intraspecific genetic variation, traditional morphological identification often fails to distinguish species accurately, particularly for closely related species such as *F. incarnatum* and *F. equiseti*, which are frequently grouped within the *F. incarnatum-equiseti* species complex (FIESC) [27,32]. Furthermore, due to high sequence conservation within the genus, single gene regions like the internal transcribed spacer (ITS) are often insufficient for delineation [24,25]. In this study, a combined phylogenetic analysis using *TEF-1α* and *RPB2* successfully identified strain Y2 as *F. incarnatum*. This corroborated previous findings that *TEF-1α* and *RPB2* are high-resolution molecular markers for species identification and phylogenetic analysis in *Fusarium*, with *RPB2* showing particular advantages in distinguishing members within the FIESC [25,27]. Based on the concatenated *TEF-1α* and *RPB2* phylogeny, strain Y2 clustered within a well-supported clade containing published *F. incarnatum* reference strains (JJ6-2 and ZL2-1) from a recent Chinese wheat crown rot survey [26]. We acknowledge that the FIESC is taxonomically challenging, with many phylogenetic species currently designated by numerical codes (e.g., FIESC 1-38) rather than formal Latin names [25,27]. Therefore, while we refer to strain Y2 as *F. incarnatum* based on current evidence and its placement within the Incarnatum clade—consistent with the identification criteria used in recent FCR pathogen diversity studies [26]—we recognize it as a member of the broader FIESC.

Given the taxonomic placement of *F. incarnatum* Y2 within the FIESC, which includes plant-pathogenic species, evaluating its biosafety was an essential first step before considering any potential application. In this study, we evaluated the pathogenicity of *F. incarnatum* Y2 on wheat stem bases using a mycelial plug inoculation assay. Our results demonstrated that strain Y2 caused no visible lesions on wheat stem bases, while the positive control *F. pseudograminearum* produced severe necrotic lesions. These findings indicate that *F. incarnatum* Y2 is non-pathogenic to wheat under the tested conditions, providing an initial biosafety indication for its potential application. However, comprehensive safety assessments—including genomic screening for mycotoxin biosynthetic gene clusters (e.g., *FUS*, *TRI*), evaluation of mycotoxin production under various culture conditions, pathogenicity testing on non-target crops, and environmental risk evaluation-remain essential before any field application can be considered.

Equisetin, isolated from the fermentation products of *F. incarnatum* Y2, is a tetramic acid derivative with a unique molecular skeleton that confers diverse biological activities [33]. Although equisetin was initially isolated from *F. equiseti* [17,18], our data confirmed that the genetic potential to produce this compound was not exclusive to that species but was also conserved within the FIESC [33], suggesting a shared or convergent evolutionary origin of the PKS-NRPS biosynthetic pathway within specific *Fusarium* clades. Furthermore, our study expanded the known antimicrobial spectra of equisetin, particularly against *Fusarium* pathogens threatening wheat production. While previous studies have confirmed its activity against *B. cinerea*, *S. sclerotiorum*, and bacterial pathogens [21,22], this study is the first to report the inhibitory effects of equisetin on *F. pseudograminearum* and *B. sorokiniana*. Notably, equisetin exhibited higher sensitivity against *F. pseudograminearum* (MIC: 16 μg/mL) compared to *F. graminearum* (MIC: 64 μg/mL). This differential sensitivity suggested variations in the target sites—possibly related to cell wall synthesis or membrane integrity—between these *Fusarium* species [5,11].

To gain deeper insights into the antifungal mechanism of equisetin, we performed transcriptomic analysis on *F. pseudograminearum* treated with 16 μg/mL equisetin. A total of 2151 differentially expressed genes (DEGs) were identified, with 785 upregulated and 1366 downregulated. Remarkably, GO enrichment analysis revealed that 103 significant terms were almost exclusively related to ribosome structure, translation, and ribosome biogenesis, and all were dominated by upregulated genes. For instance, “cytosolic ribosome” (GO:0022626) and “cytoplasmic translation” (GO:0002181) showed 100% upregulation among the DEGs within these terms. KEGG pathway analysis further confirmed that the ribosome pathway (map03010) was the most significantly enriched, containing 74 DEGs with 71 upregulated. These results indicated that equisetin induced a polarized transcriptomic response: a strong and selective activation of the entire ribosome life cycle—from pre-ribosome assembly and rRNA processing to nuclear export and cytoplasmic translation—while simultaneously causing widespread downregulation of non-core functional genes, including nitrogen metabolism (map00910), which was the only significantly enriched pathway dominated by downregulated genes. The reliability of these findings was confirmed by qRT-PCR validation of seven representative genes, including four ribosomal protein genes (*FpRPL2*, *FpRPL24*, *FpRPS0*, and *FpRPS27a*) and three nitrogen metabolism genes (*FpCA2*, *FpGLN1*, and *FpGDH2*), all of which showed expression trends consistent with the RNA-seq data. However, whether this transcriptional reprogramming represents a direct molecular target of equisetin or a secondary compensatory response remains to be determined. The ribosomal activation pattern observed in our study is mechanistically distinct from previously proposed mechanisms of equisetin action. Han et al. (2025) demonstrated that equisetin disrupts membrane integrity in *Enterococcus faecalis* [34], while Zhong et al. (2024) reported that equisetin inhibits adipocyte differentiation through AMPK-dependent mitochondrial regulation [35]. It is possible that equisetin exerts species-specific effects or acts through multiple targets in a concentration-dependent manner. Alternatively, the strong upregulation of ribosomal genes may represent a secondary stress response to cellular damage (e.g., membrane or mitochondrial impairment), rather than a direct interaction with the translation machinery. This hypothesis is supported by the observation that similar ribosomal stress responses have been documented in fungi treated with other antimicrobial agents [36]. We therefore interpret these findings as a transcriptomic response rather than definitive evidence of a specific mode of action.

Beyond its agricultural applications, equisetin has garnered attention for its other bioactivities. Previous studies have shown that equisetin can exert anti-obesity effects by targeting 11β-hydroxysteroid dehydrogenase type 1 (11β-HSD1) [20] and inhibit HIV-1 integrase [19], illustrating its multifunctionality in the pharmaceutical field. Several limitations of this study should be acknowledged. First, all antifungal activity assessments were performed in vitro, and no greenhouse or field trials were conducted to demonstrate disease suppression in wheat. Second, the mechanistic interpretation is based primarily on transcriptional profiling; while qRT-PCR validated the expression trends, these data do not conclusively prove that ribosomes are the direct molecular target of equisetin. Third, although strain Y2 was shown to be non-pathogenic to wheat, comprehensive biosafety evaluation remains necessary. Fourth, the minimum fungicidal concentration (MFC) of equisetin was not determined in this study, as our primary focus was on inhibitory activity. Future studies should prioritize in planta efficacy testing, phytotoxicity evaluation, formulation development, targeted functional assays, and comprehensive biosafety assessment to determine the practical feasibility of equisetin-based applications.

## 5. Conclusions

In this study, we successfully identified the endophytic strain Y2 as a member of the *F. incarnatum–equiseti* species complex (FIESC) and isolated the bioactive metabolite equisetin. Pathogenicity assessment confirmed that *F. incarnatum* Y2 is non-pathogenic to wheat, providing an initial biosafety indication for its potential application. Equisetin exhibited potent in vitro antifungal activity against major pathogens of wheat root and crown rot, particularly *F. pseudograminearum* (MIC: 16 μg/mL). Transcriptomic analysis revealed that equisetin induces a polarized transcriptional response characterized by strong activation of ribosome-related pathways and concurrent suppression of nitrogen metabolism, with qRT-PCR validation confirming the reliability of the data. While these results highlight equisetin as a promising lead compound for antifungal development, the in vitro nature of this study necessitates further in planta efficacy and safety evaluations before practical biocontrol applications can be considered.

## Figures and Tables

**Table 1:** Amplification sites, primer names, primer sequences and references used in this study.

Locus	Primer Name	Primer Sequence (5′-3′)	References
*TEF-1α*	EF1	ATGGGTAAGGARGACAAGAC	[22]
	EF2	GGARGTACCAGTSATCATGTT	
*RPB2*	5f2	GGGGWGAYCAGAAGAAGGC	[23]
	7cr	CCCATRGCTTGYTTRCCCAT	

**Table 2:** Isolates included in the phylogenetic analysis and their GenBank accession numbers.

Isolate	Species	GenBank Accession No.	
		TEF-1α	RPB2
QP1-1	*F. pseudograminearum*	OP105186	OP785247
LY1-1	*F. pseudograminearum*	OP105184	OP785245
JL10-1	*F. graminearum*	OP105199	OP785260
DN2-3	*F. graminearum*	OP105196	OP785257
HY6-1	*F. sinensis*	OP105207	OP785268
DP1-1	*F. sinensis*	OP105206	OP785267
LS1-1	*F. acuminatum*	OP105209	OP785270
YZ1-3	*F. acuminatum*	OP105210	OP785271
JJ6-2	*F. incarnatum*	OP105211	OP785272
ZL2-1	*F. incarnatum*	OP105212	OP785273
JWS1-1	*F. ipomoeae*	OP105213	OP785274
NRRL 43640	*F. ipomoeae*	GQ505667	GQ505845
QL1-1	*F. flocciferum*	OP105214	OP785275
NRRL 40008	*F. flocciferum*	OL772897	OL773201
WC1-5	*F. proliferatum*	OP105215	OP785276
NRRL 62905	*F. proliferatum*	MN193865	MN193893
WR3-2	*F. asiaticum*	OP105216	OP785277
NRRL 13818	*F. asiaticum*	MW233069	MW233412
LS1-3	*F. culmorum*	OP105217	OP785278
NRRL 52792	*F. culmorum*	JF740860	JF741186
JL3-3	*F. oxysporum*	OP105218	OP785279
NRRL 25378	*F. oxysporum*	HM347116	HM347208
NRRL 20697	*F. equiseti*	GQ505594	GQ505772
NRRL 26419	*F. equiseti*	GQ505599	GQ505777
CBS 191.86	*S. vesicarium*	KC584731	KC584471

**Table 3:** MICs of equisetin against three plant-pathogenic fungi (µg/mL).

Treatment	*B. sorokiniana*	*F. pseudograminearum*	*F. graminearum*
Equisetin	32	16	64
Crude extract	128	128	128
Carbendazim	8	8	8

## Data Availability

The raw RNA-seq data have been deposited in the NCBI Sequence Read Archive (SRA) under accession number PRJNA1483097. The HR-ESI-MS and NMR spectra data supporting the findings of this study are available in the Supplementary Materials. Other data presented in this study are available from the corresponding authors upon reasonable request.

## Supplementary Materials

The following supporting information can be downloaded at: https://www.mdpi.com/article/10.3390/genes17080869/s1, Figure S1: HPLC analysis of the crude extract; Figure S2: HPLC analysis of equisetin prior to NMR detection; Figure S3: HR-ESI-MS of equisetin; Figure S4: The ^1^H NMR spectrum of **1**; Figure S5: The ^13^C NMR spectrum of **1**; Table S1: qRT-PCR primers used in this study; Table S2: GO enrichment analysis of differentially expressed genes in Fusarium pseudograminearum treated with equisetin.
