# Supplementary material for: Antifungal Activity and Transcriptomic Profiling of Equisetin from Endophytic Fusarium incarnatum Y2 Against Major Wheat Root and Crown Rot Pathogens
Source: Genes (Basel). 2026 Jul 25;17(8):869. doi: 10.3390/genes17080869 (PMC13512544; doi:10.3390/genes17080869)
Supplement: Supplementary file 1 [file genes-17-00869-s001.zip › genes-4369445-supplementary.pdf]

Attached Materials

Antifungal Activity and Transcriptomic Profiling of Equisetin from Endophytic *Fusarium incarnatum* Y2 Against Major Wheat Root and Crown Rot Pathogens

Miao Liu [1,2], Feifan Wang [1], Luying Han [1], Feiyu Yan [3], Yinshan Huang [1], Yuehua Geng [3,4], Chunnan Wen [1,2], Luyang Song [3], Meng Zhang [3,4], Fang Liu [5,*] and Qingzhou Ma [3,4,*]

1 College of Agronomy, Henan Agricultural University, Zhengzhou 450046, China; liumiao@henau.edu.cn (M.L.); w13949147723@163.com (F.W.); 18838280398@163.com (L.H.); xhh1479@163.com (Y.H.); cnwen@henau.edu.cn (C.W.)

2 Zhengzhou Key Laboratory of Germplasm Resources Innovation and Metabolic Engineering of Traditional Chinese Medicine, Henan Agricultural University, Zhengzhou 450046, China

3 College of Plant Protection, Henan Agricultural University, Zhengzhou 450046, China; 17630711375@163.com (F.Y.); gyh@henau.edu.cn (Y.G.); lysong@henau.edu.cn (L.S.); zm2006@126.com (M.Z.)

4 State Key Laboratory of High-Efficiency Production of Wheat-Maize Double Cropping, Henan Agricultural University, Zhengzhou 450002, China

5 College of Resources and Environment, Henan Agricultural University, Zhengzhou 450046, China

* Correspondence: f-liu113@163.com (F.L.), 15237171177@163.com (Q.M.)

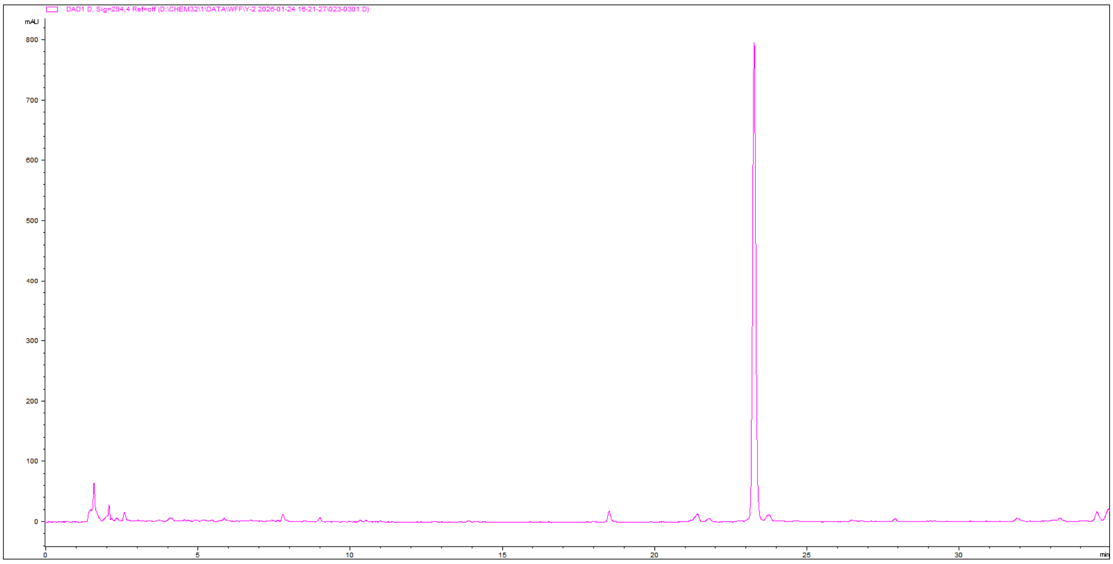

Figure S1 HPLC analysis of the crude extract.

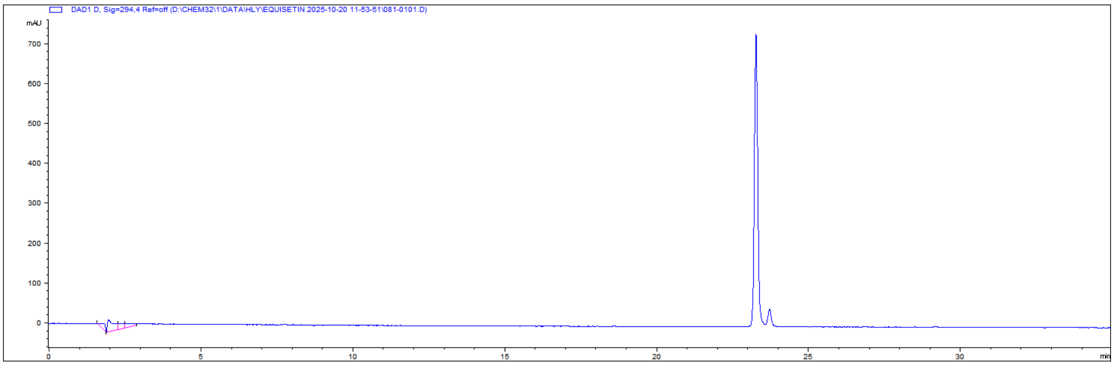

Figure S2 HPLC analysis of equisetin prior to NMR detection.

Note: HPLC analysis for **Figure S1** and **Figure S2** were performed on an Agilent 1100 equipped with a Zorbax SB-C8 (4.6 mm × 150 mm) column at a flow rate of 1 mL/min. The elution conditions were set as: 0-30 min, 10%-100% acetonitrile/H_2O; 30-35 min, 100% acetonitrile. The detect wavelength was 294 nm.

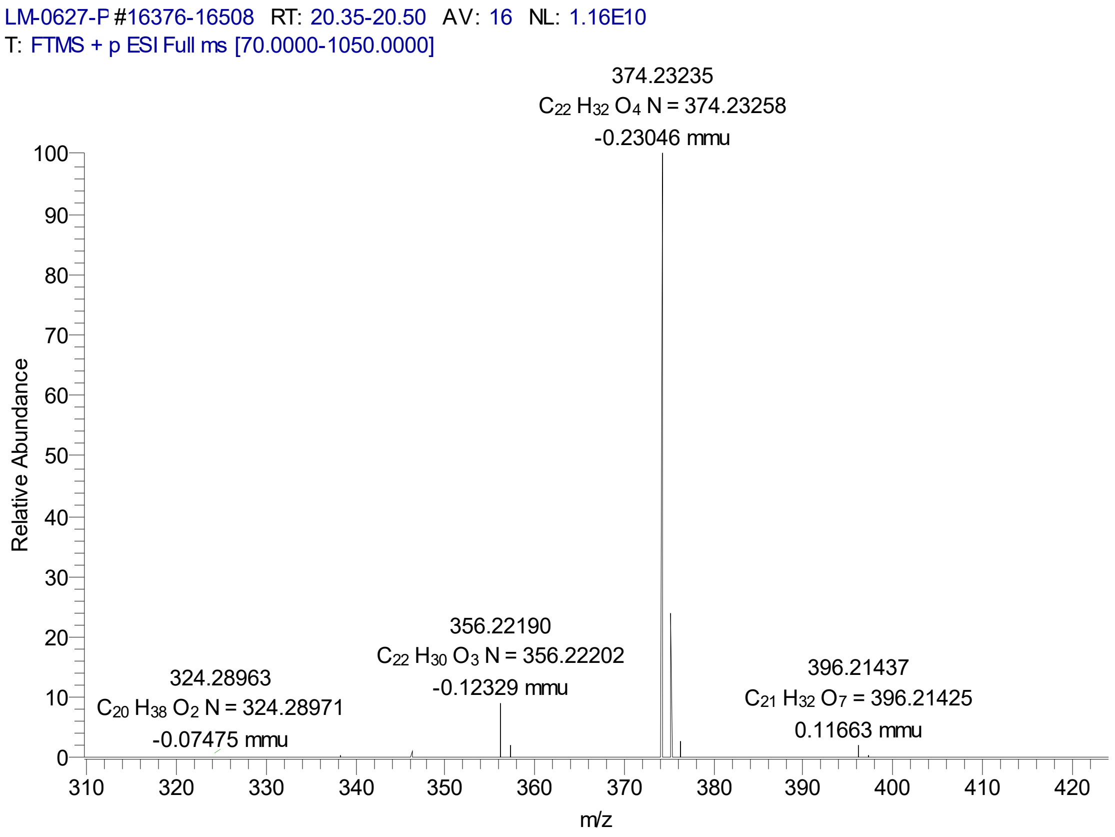

Figure S3 HR-ESI-MS of equisetin.

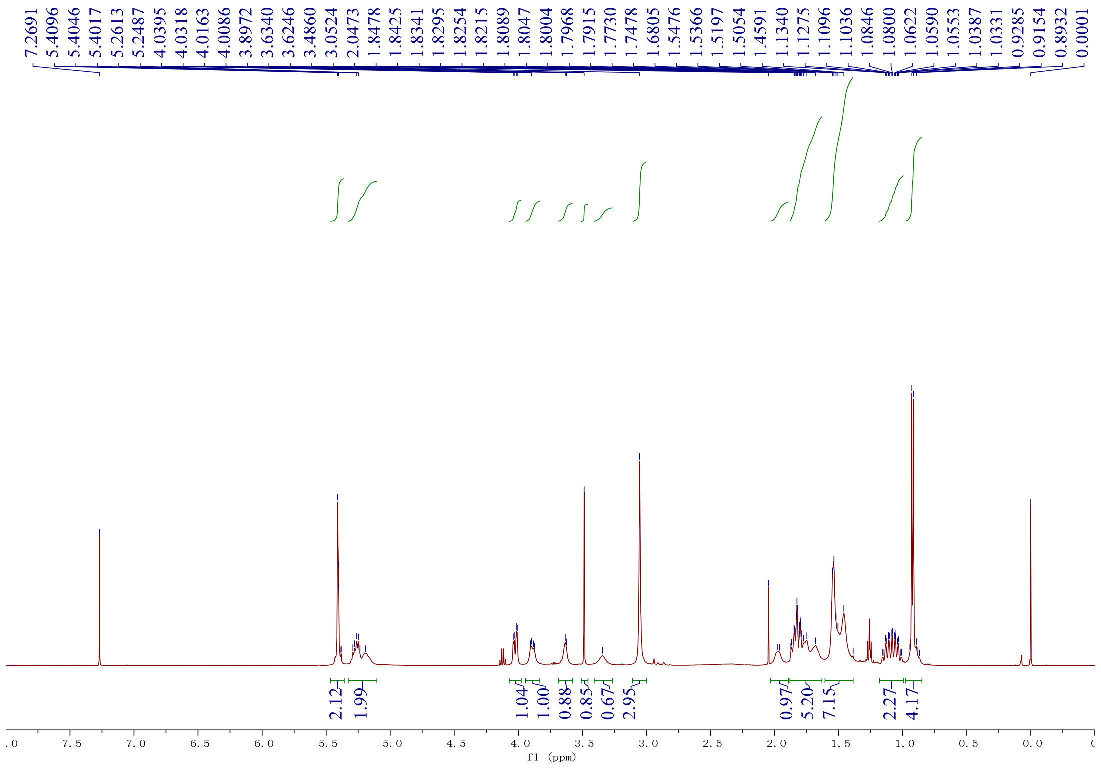

Figure S4 The ^{1}H NMR spectrum of **1**.

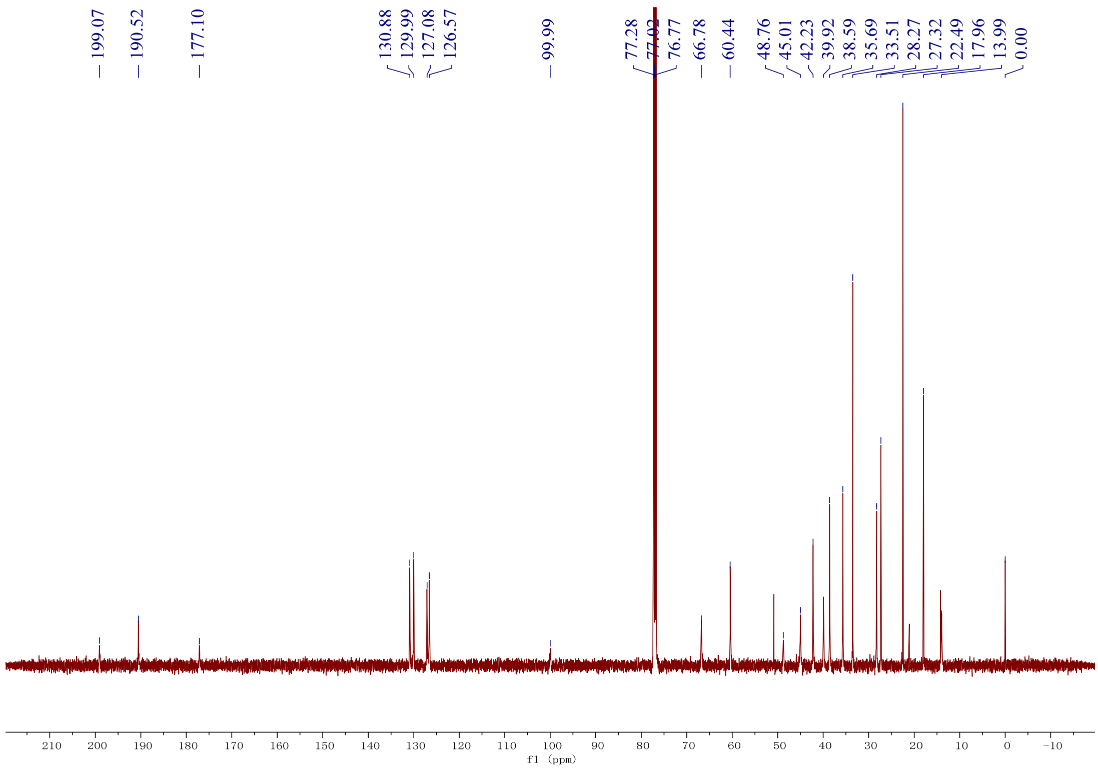

Figure S5 The ^{13}C NMR spectrum of **1**.

Table S1. qRT-PCR primers used in this study

Name	Sequence (5′-3′)	Purpose
FpRPL2-F	CCAAGGGTGGTCGTAATGTGT	Expression of *FpRPL2*
FpRPL2-R	GCAAGAATGTAGGTGTGACGC	
FpRPL24-F	AGCGTGGTATCGTTGGTGC	Expression of *FpRPL24*
FpRPL24-R	ACCCTGCTTGCTGACCTGT	
FpRPS0-F	TGTCATCTCTGCCCGTCCCT	Expression of *FpRPS0*
FpRPS0-R	CGTGGCGACCCTTGTTGTT	
FpRPS27a-F	CAGGACAAGGAGGGTATCCC	Expression of *FpRPS27a*
FpRPS27a-R	ACCAAGTGAAGGGTGGACTC	
FpCA2-F	CGGCGAGGCATTTATCCAC	Expression of *FpCA2*
FpCA2-R	TCAGCAGACCCAAGTCCTG	
FpGLN1-F	CCGATGACAGACCCTATGGC	Expression of *FpGLN1*
FpGLN1-R	GCGGTGAAGGAAGAAGCGA	
FpGDH2-F	TTGGTGGCTCCGTTGTCTC	Expression of *FpGDH2*
FpGDH2-R	TCCTCCTTGCTGACCTCGT	
RT-FpTEF1α-F	TCACCACTGAAGTCAAGTCC	Internal reference primers of *F. pseudograminearum*
RT-FpTEF1α-R	ACCAGCGACGTTACCACGTC	

Table S2. GO enrichment analysis of differentially expressed genes in *Fusarium pseudograminearum* treated with equisetin.

GO_ID	Category	Term	Up	Down	DEG	Total	Pvalue	AdjustPvalue
GO:0022626	CC	cytosolic ribosome	69	0	69	92	1.7721e-31	4.7478e-28
GO:0002181	BP	cytoplasmic translation	107	0	107	189	1.83101e-31	4.7478e-28
GO:0005840	CC	ribosome	83	0	83	152	5.68107e-23	9.82067e-20
GO:0003735	MF	structural constituent of ribosome	69	0	69	116	4.36616e-22	5.43157e-19
GO:0044391	CC	ribosomal subunit	73	0	73	128	8.62242e-22	7.45264e-19
GO:0022625	CC	cytosolic large ribosomal subunit	36	0	36	42	1.9066e-20	1.41252e-17
GO:0043603	BP	cellular amide metabolic process	150	18	168	476	1.40615e-18	9.11537e-16
GO:0006518	BP	peptide metabolic process	136	5	141	382	1.97904e-17	1.14037e-14
GO:0043604	BP	amide biosynthetic process	141	6	147	411	9.55943e-17	4.95752e-14
GO:0043043	BP	peptide biosynthetic process	130	1	131	355	3.6825e-16	1.73613e-13
GO:0006412	BP	translation	125	0	125	339	2.18837e-15	9.4574e-13
GO:1901566	BP	organonitrogen compound biosynthetic process	206	28	234	796	9.20869e-15	3.67356e-12
GO:0042254	BP	ribosome biogenesis	105	2	107	289	2.41775e-13	8.95603e-11
GO:0030684	CC	preribosome	64	1	65	145	1.03114e-12	3.426e-10
GO:0022627	CC	cytosolic small ribosomal subunit	29	0	29	41	1.057e-12	3.426e-10
GO:0015934	CC	large ribosomal subunit	40	0	40	71	3.71381e-12	1.13293e-09

GO:0015935	CC	small ribosomal subunit	33	0	33	58	2.13783e-10	6.15933e-08
GO:1990904	CC	ribonucleoprotein complex	147	3	150	495	2.82028e-10	7.69787e-08
GO:0005198	MF	structural molecule activity	71	2	73	195	1.32038e-09	3.42374e-07
GO:0044281	BP	small molecule metabolic process	123	64	187	669	2.08009e-09	5.13683e-07
GO:0022613	BP	ribonucleoprotein complex biogenesis	115	3	118	376	3.54166e-09	8.34866e-07
GO:0030687	CC	preribosome, large subunit precursor	32	1	33	66	1.77529e-08	4.00289e-06
GO:0005829	CC	cytosol	140	25	165	590	2.58255e-08	5.3175e-06
GO:0043436	BP	oxoacid metabolic process	80	34	114	371	2.66593e-08	5.3175e-06
GO:0006082	BP	organic acid metabolic process	80	34	114	372	3.15693e-08	6.06364e-06
GO:0019752	BP	carboxylic acid metabolic process	78	33	111	361	4.05013e-08	7.50141e-06
GO:0042274	BP	ribosomal small subunit biogenesis	47	0	47	115	6.15633e-08	1.10092e-05
GO:0034660	BP	ncRNA metabolic process	113	7	120	403	8.09638e-08	1.39766e-05
GO:0006520	BP	cellular amino acid metabolic process	57	16	73	212	8.35472e-08	1.39766e-05
GO:0042273	BP	ribosomal large subunit biogenesis	37	1	38	88	2.1642e-07	3.50736e-05
GO:0006364	BP	rRNA processing	69	1	70	210	6.56382e-07	0.000103151
GO:0006418	BP	tRNA aminoacylation for protein translation	20	0	20	36	1.44575e-06	0.000220519

GO:0005832	CC	chaperonin-containing T-complex	8	0	8	8	1.97449e-06	0.000292563
GO:0005852	CC	eukaryotic translation initiation factor 3 complex	11	0	11	14	2.86756e-06	0.000409513
GO:0006458	BP	'de novo' protein folding	13	1	14	21	2.92171e-06	0.000409513
GO:0044283	BP	small molecule biosynthetic process	67	28	95	322	3.55824e-06	0.000485607
GO:0000462	BP	maturation of SSU-rRNA from tricistronic rRNA transcript (SSU-rRNA, 5.8S rRNA, LSU-rRNA)	34	0	34	83	4.08261e-06	0.000537349
GO:0004812	MF	aminoacyl-tRNA ligase activity	19	0	19	35	4.24823e-06	0.000537349
GO:0016875	MF	ligase activity, forming carbon-oxygen bonds	19	0	19	35	4.24823e-06	0.000537349
GO:0043038	BP	amino acid activation	20	0	20	38	4.46665e-06	0.000538699
GO:0043039	BP	tRNA aminoacylation	20	0	20	38	4.46665e-06	0.000538699
GO:0034470	BP	ncRNA processing	88	5	93	316	5.13228e-06	0.00060491
GO:0042255	BP	ribosome assembly	25	1	26	57	5.46029e-06	0.000629268
GO:0016072	BP	rRNA metabolic process	70	3	73	235	7.38421e-06	0.000827725
GO:0030490	BP	maturation of SSU-rRNA	36	0	36	92	7.50155e-06	0.000827725
GO:0097064	BP	ncRNA export from nucleus	13	2	15	25	8.6735e-06	0.0009371
GO:0000054	BP	ribosomal subunit export from nucleus	17	1	18	34	1.21985e-05	0.00124042
GO:0033750	BP	ribosome	17	1	18	34	1.21985e-05	0.00124042

		localization						
GO:0071166	BP	ribonucleoprotein complex localization	32	2	34	88	1.85614e-05	0.00180862
GO:1901615	BP	organic hydroxy compound metabolic process	22	22	44	125	1.88325e-05	0.00180862
GO:0006405	BP	RNA export from nucleus	32	2	34	89	2.45817e-05	0.00231783
GO:0051082	MF	unfolded protein binding	23	2	25	58	2.78498e-05	0.00257909
GO:0003674	MF	molecular_ function	427	210	637	3089	3.15389e-05	0.00286949
GO:0016874	MF	ligase activity	27	7	34	90	3.23335e-05	0.00289106
GO:0008652	BP	cellular amino acid biosynthetic process	29	10	39	109	3.69433e-05	0.00324725
GO:1901607	BP	alpha-amino acid biosynthetic process	26	10	36	99	4.95915e-05	0.00428636
GO:0050658	BP	RNA transport	33	2	35	96	5.92555e-05	0.00495643
GO:0051236	BP	establishment of RNA localization	33	2	35	96	5.92555e-05	0.00495643
GO:0006611	BP	protein export from nucleus	33	3	36	100	6.34138e-05	0.00522006
GO:0005730	CC	nucleolus	61	6	67	224	6.90184e-05	0.00559265
GO:0006913	BP	nucleocytoplasmic transport	42	4	46	139	7.23693e-05	0.0056182
GO:0051169	BP	nuclear transport	42	4	46	139	7.23693e-05	0.0056182
GO:0016491	MF	oxidoreductase activity	39	36	75	258	7.25837e-05	0.0056182
GO:0050657	BP	nucleic acid transport	33	2	35	97	7.58764e-05	0.00578669
GO:0051168	BP	nuclear export	33	3	36	101	8.06419e-05	0.00600036
GO:0006407	BP	rRNA export from nucleus	10	0	10	15	8.44203e-05	0.00600036
GO:0051029	BP	rRNA transport	10	0	10	15	8.44203e-05	0.00600036
GO:0008204	BP	ergosterol metabolic process	12	6	18	38	8.44632e-05	0.00600036
GO:0016128	BP	phytosteroid metabolic process	12	6	18	38	8.44632e-05	0.00600036

GO:0000027	BP	ribosomal large subunit assembly	15	1	16	33	0.000148721	0.0104226
GO:1901617	BP	organic hydroxy compound biosynthetic process	14	16	30	82	0.00018504	0.0127949
GO:0044107	BP	cellular alcohol metabolic process	12	6	18	40	0.000192435	0.0131312
GO:0016053	BP	organic acid biosynthetic process	42	13	55	181	0.000200035	0.0132997
GO:0046394	BP	carboxylic acid biosynthetic process	42	13	55	181	0.000200035	0.0132997
GO:0006696	BP	ergosterol biosynthetic process	12	5	17	37	0.000212335	0.0134289
GO:0016129	BP	phytosteroid biosynthetic process	12	5	17	37	0.000212335	0.0134289
GO:0044108	BP	cellular alcohol biosynthetic process	12	5	17	37	0.000212335	0.0134289
GO:0097384	BP	cellular lipid biosynthetic process	12	5	17	37	0.000212335	0.0134289
GO:0030686	CC	90S preribosome	27	0	27	72	0.000241613	0.0150964
GO:0140101	MF	catalytic activity, acting on a tRNA	26	2	28	76	0.000263519	0.0162691
GO:0006066	BP	alcohol metabolic process	17	16	33	96	0.000347211	0.021184
GO:0046165	BP	alcohol biosynthetic process	13	13	26	70	0.000374478	0.022538
GO:0003824	MF	catalytic activity	234	148	382	1753	0.00038212	0.022538
GO:0003743	MF	translation initiation factor activity	15	0	15	32	0.000382442	0.022538
GO:1902653	BP	secondary alcohol biosynthetic	12	6	18	42	0.00040531	0.0236173

		process						
GO:0008202	BP	steroid metabolic process	12	8	20	49	0.000427324	0.0246234
GO:0061077	BP	chaperone-mediated protein folding	11	1	12	23	0.000434202	0.0247447
GO:1901564	BP	organonitrogen compound metabolic process	264	93	357	1628	0.000443371	0.0249926
GO:0044183	MF	protein folding chaperone	8	0	8	12	0.000462455	0.0255137
GO:0101031	CC	chaperone complex	8	0	8	12	0.000462455	0.0255137
GO:0016125	BP	sterol metabolic process	12	7	19	46	0.000495921	0.02679
GO:1902652	BP	secondary alcohol metabolic process	12	7	19	46	0.000495921	0.02679
GO:0019843	MF	rRNA binding	16	0	16	36	0.000518338	0.0277124
GO:0071941	BP	nitrogen cycle metabolic process	3	7	10	18	0.000695787	0.036662
GO:0000056	BP	ribosomal small subunit export from nucleus	6	1	7	10	0.000706941	0.036662
GO:0006551	BP	leucine metabolic process	7	0	7	10	0.000706941	0.036662
GO:0006457	BP	protein folding	25	4	29	84	0.000723778	0.036892
GO:0006450	BP	regulation of translational fidelity	11	0	11	21	0.000725605	0.036892
GO:0016614	MF	oxidoreductase activity, acting on CH-OH group of donors	11	12	23	62	0.000823382	0.0414569
GO:0003723	MF	RNA binding	96	9	105	412	0.000853711	0.0425707
GO:0000470	BP	maturation of LSU-rRNA	17	0	17	41	0.00092511	0.0456916
GO:0006403	BP	RNA localization	34	2	36	113	0.000981857	0.0480369
GO:0071540	CC	eukaryotic translation	6	0	6	8	0.00103475	0.0496873

		initiation factor 3 complex, eIF3e						

Note: GO_ID, Gene Ontology identifier; Category, functional category (CC: cellular component, BP: biological process, MF: molecular function); Term, description of the GO term; Up, number of upregulated genes; Down, number of downregulated genes; DEG, total number of differentially expressed genes; Total, total number of background genes annotated to this GO term; Pvalue, P-value from enrichment analysis; adjustPvalue, P-value adjusted for multiple hypothesis testing (FDR).
